# Long term survival in three pancreatic cancer patients following Whipple’s procedure and allogeneic hematopoietic cell transplantation

**DOI:** 10.3389/fimmu.2026.1766025

**Published:** 2026-06-23

**Authors:** Olle Ringden, Behnam Sadeghi, Johan Törlen, Stephan Mielke, Johan Permert

**Affiliations:** 1 Translational Cell Therapy Research, Department of Clinical Science Intervention and Technology (CLINTEC), Karolinska institute, Stockholm, Sweden; 2 Department of Cell Therapy and Allogeneic Stem Cell Transplantation (CAST), Karolinska Comprehensive Cancer Center, Karolinska Advanced Therapy Medicinal Products (ATMP) Center, Karolinska University Hospital, Stockholm, Sweden; 3 Therapeutic immunology and transfusion medicine, Department of Clinical Science, Intervention and Technology (CLINTEC), Karolinska Institute, Stockholm, Sweden; 4 Department of Laboratory Medicine, Karolinska Institute, Stockholm, Sweden; 5 Department of Surgery, CLINTEC, Karolinska Institutet, Stockholm, Sweden

**Keywords:** graft versus tumor effect (GvT), hematopoietic cell transplantation, pancreatic ductal carcinoma, survival, Whipple’s surgery

## Abstract

**Introduction:**

Pancreatic ductal adenocarcinoma (PDAC) is associated with an extremely poor prognosis, with long-term survival being exceedingly rare. Here, we follow-up on three long-term survivors.

**Methods:**

This study initially included eight patients with PDAC who underwent radical resection via Whipple’s procedure with no evidence of distant metastases. Two of these patients received allogeneic hematopoietic cell transplantation (HCT) in a previously published trial. The remaining six patients, who served as controls and did not receive HCT, died of progressive PDAC within one to four years. Additionally, a 60-year-old male with PDAC who underwent partial pancreatectomy and gastrectomy received HCT from a matched unrelated donor.

**Results:**

Patient 1 developed mild acute graft-versus-host disease (GVHD) of the skin, followed by chronic GVHD. He subsequently underwent surgery for oral cancer and remains alive 21 years after the initial PDAC diagnosis, with no evidence of recurrence. Patient 2 experienced severe acute gastrointestinal GVHD and recurrent chronic bronchiolitis obliterans. He died of esophageal cancer 19 years after PDAC diagnosis. Patient 3 experienced graft rejection and multiple medical complications, including radical surgery for colorectal cancer, yet remains cancer-free 15 years after PDAC diagnosis.

**Discussion:**

In conclusion, HCT may confer anticancer effects in patients with radically resected PDAC. Further investigations are warranted to confirm its therapeutic value.

ClinicalTrials.gov Identifier: NCT02207985

## Introduction

Pancreatic ductal adenocarcinoma (PDAC) is considered a neglected malignancy due to its extremely limited therapeutic options and dismal prognosis, reflected in persistently low five-year survival rates (1). Although genetically diverse cancers may, in principle, respond to immunotherapy, all attempts in PDAC have thus far been disappointing (2). The median overall survival remains approximately four months (3, 4). PDAC ranks among the leading causes of cancer-related death worldwide, with a steadily increasing incidence. Despite the generally poor outcomes in PDAC, modest progress has been achieved: the five-year survival rate has increased from 4% to 23% over the past three decades (3). This improvement is mainly attributable to advances in oncologic management, including refined surgical techniques and the centralization of patient care (5–7).

Implementing precision oncology approaches in PDAC remain particularly challenging (8). Immunomodulatory strategies—such as checkpoint inhibitors, Ibrutinib, niraparib, anti-PD-1, anti-PD-L1, nivolumab, ipilimumab, and PARP inhibitors—either alone or combined with chemotherapy and tumor-infiltrating lymphocytes (TILs), have so far failed to demonstrate major clinical success (9, 10).

Hematopoietic cell transplantation (HCT) is a well-established therapeutic modality for hematologic malignancies, particularly acute leukemia (11). A major adverse effect of allogeneic HCT is graft-versus-host disease (GVHD), which can manifest in both acute and chronic forms. Notably, GVHD—especially chronic GVHD—exerts potent anti-leukemic activity (12, 13).

Graft-versus-tumor (GVT) effects have also been reported in solid tumors, including breast carcinoma (14, 15), renal cell carcinoma (16), colon carcinoma (17) and ovarian carcinoma (18). HCT has been attempted in patients with pancreatic carcinoma, suggesting the presence of a graft-versus-pancreatic cancer effect, although none of these PDAC patients achieved long-term survival (19–21). Based on the potential graft-versus-solid tumor mechanism, large single-center and multicenter studies have been conducted using HCT primarily in patients with renal cell carcinoma and colorectal carcinoma (22–24). However, most of these patients had widespread disease at the time of transplantation, and recurrent or persistent cancer remained the most common cause of death (25). HCT has a well-documented anti-cancer effect, which apart from hematological malignancies also was documented in solid tumors including PDAC. Therefore, we thought it appropriate to select radically operated PDAC patients with no visible tumor burden to use the potent anticancer effect of HCT. We presented and discussed the project twice before we got approval from the ethical committee of Karolinska Institutet (2006/858-32/4). The allogeneic graft-vs -tumor (GVT)- effect following HCT is strongly linked to acute and especially chronic GVHD (12, 13). GVHD and the GVT- effect are induced by alloreactive cytotoxic T-cells. Cytotoxic T-cell associated protein-4 (CTLA-4), is a negative regulator of T- cell function, a so-called checkpoint inhibitor which was used to treat PDAC. However, it inhibits T cell activation and is used to prevent GVHD. It is registered in the US for unrelated donor HCT (26). If the use of CTLA-4 by preventing GVHD, may abrogate the GVT- effect is possible, but not established. Given these findings, we sought to evaluate HCT as an adjuvant immunotherapeutic approach for patients with radically resected PDAC and no distant metastases. Here, we report updated long-term outcomes of consolidative allogeneic immunotherapy using HCT based on biological selection, in which transplantation was offered only to patients with an available HLA-matched related donor within a clinical trial (NCT02207985). Ethical approval for extended follow-up was recently obtained (DNR 2023-06298-01) to reassess three previously reported PDAC patients who underwent HCT following Whipple’s procedure (25, 27).

## Materials and methods

### Patients

Two male patients, aged 47 and 66 years, who underwent radical Whipple’s procedure, were offered HCT because they had HLA-identical sibling donors (**Table 1**, Patient Characteristics). The older patient was a heavy smoker with a history of chronic obstructive pulmonary disease (COPD). Both patients received non-myeloablative conditioning consisting of fludarabine and cyclophosphamide, combined with cyclosporine A and methotrexate. A third patient, a 60-year-old male with PDAC who underwent radical surgery including partial gastrectomy, also received HCT.

**Table 1:** Patients characteristics and summary.

Patient no	Sex	Age at HCT	HCT donor	Donor age	PDAC stage	Metastasis	Conditioning	Immune suppression	GVHD		Secondary malignancy	CA19-9	CT Scan	Survival from diagnosis
									acute	chronic				
1	M	46	MRD	45	T3N0V1PN1	0	Flu/Cy	CyA/Mtx	I	I	Oral cancer	Neg	Neg	Alive 21yrs
2	M	57	MRD	54	T3N1V1PN1	0	Flu/Cy	CyA/Mtx	III	II	Esophageal cancer	Neg	Neg	Dead 19yrs
3	M	60	MUD	46	T3N1V1PN1	0	Flu/Cy	Mtx/CyA ATG	Graft rejection		Colorectal & Skin cancer	Neg	Neg	Alive 15yrs

UPN, unique patients number; HCT, hematopoietic cell transplantation; GVHD, graft versus host diseases; MRD, matched related donor; MUD, matched unrelated donor; Flu, fludarabine; Cy, Cyclophosphamide; CyA, cyclosporine; Mtx, methotrexate; ATG, antithymocyte globulin; Neg, Negative.

The six control patients were informed of the trial, and agreed, but did not have HLA -identical sibling donors. These patients were the same ages as the two HCT recipients and had complete resection, R0/R1. All six were T3, three N1 and three N0, five V1 and one V0 and all Pn 1. There was one female and five males. Three control patients were treated with Gemcitabine, and one was given 5-Fu. Two of the controls declined to receive chemotherapy. Survival was from 16 to 48 months (median 35 months) from pancreatectomy. The patients were previously reported in detail (25, 27). Data of final analysis: January 2024.

### Donors and grafts

High-resolution PCR-based HLA typing was performed for both HLA class I and class II alleles. The first two patients were included because they had matched related donors (MRDs), both being HLA-identical siblings. For Patient 3, an HLA-matched unrelated donor (MUD) was identified, with compatibility at 9 out of 12 alleles for HLA-A, -B, -C, -DR, -DP, and -DQ.

All patients received mobilized peripheral blood stem cells collected from donors after granulocyte colony-stimulating factor (G-CSF) administration (10 μg/kg/day; Neupogen, Amgen, Stockholm) (28).

### Conditioning and immunosuppression

The conditioning regimen consisted of fludarabine (30 mg/m²/day for five days) followed by cyclophosphamide (60 mg/kg/day for two days). Post-transplant immunosuppression included cyclosporine A (CyA), administered orally at 3 mg/kg/day in two divided doses. For recipients of MRD grafts, serum CyA concentrations were maintained around 100 ng/mL. In the patient receiving a MUD graft, CyA levels were maintained between 200 and 300 ng/mL. CyA was given at full dose for three months and tapered thereafter, with discontinuation at six months in the absence of GVHD.

Methotrexate was administered on days +1 (15 mg/m²) and on days +3, +6, and +11 (10 mg/m²) (29). The MUD recipient additionally received anti-thymocyte globulin (ATG; SangStat, Lyon, France) at a total dose of 8 mg/kg, given on days −2, −1, and 0 before HCT. Acute GVHD was initially treated with prednisolone at a dose of 1 mg/kg/day.

### Cancer screening

The tumor marker CA 19–9 was measured monthly during the first year after HCT and every three months thereafter. Ca19–9 levels were followed monthly during the first three to five years after HCT and then yearly or sometimes also at visits to the outpatient clinic. Multidetector computed tomography (CT) scans of the thorax and abdomen were performed annually.

### Supportive care

Infection prophylaxis included ciprofloxacin (oral), fluconazole (oral), nystatin (oral), acyclovir (oral), and co-trimoxazole, administered according to our institutional HCT protocol (30). Patients 1 and 3 were treated in reversed isolation during the pancytopenic phase, whereas Patient 2 was managed at home (31, 32).

### Controls

A comparative analysis included six control patients (grade T3 for all; N1: 3 cases; N0: 3 cases; V1: 5 cases; V0: 1 case; Pn1: all) who underwent Whipple’s operation but did not receive HCT because of the absence of HLA-identical sibling donors.

Adjuvant treatment varied: four patients received gemcitabine or 5-fluorouracil, while two declined chemotherapies. Additionally, a 60-year-old male (Patient 3, T3N1V1Pn1) who underwent partial pancreatectomy and partial gastrectomy received HCT from a matched unrelated donor. All patients achieved complete histological resection (R0/R1).

### Ethical approvals

Ethical approval for the study was granted by the Karolinska Institutet (88/96, 69/99, 385/99, 2006/858-324, 2023-06298-01), and the study was registered at ClinicalTrials.gov (NCT02207985) (25, 27).

## Results

All patients who underwent pancreatectomy developed diabetes mellitus. The control group, which did not receive HCT, died of progressive PDAC within a median of three years (range: one to three years).

Patient 1 developed mild acute graft-versus-host disease (GVHD) of the skin, which responded well to corticosteroid therapy, followed by chronic GVHD affecting the oral and gastrointestinal mucosa. The chronic GVHD was successfully managed with photopheresis. Ten years after HCT, he underwent surgery for oral cancer. At present—twenty-one years after the initial PDAC diagnosis—he remains alive with no evidence of PDAC recurrence.

Patient 2 developed grade III gastrointestinal acute GVHD, which was responsive to steroids, along with recurrent exacerbations of chronic obstructive pulmonary disease (COPD) requiring home oxygen therapy. He later developed esophagitis and subsequently esophageal cancer, which led to his death at home nineteen years after the diagnosis of PDAC.

Patient 3 This patient with PDAC, contacted us because he heard of the study and wanted to undergo HCT. Since he had no HLA - identical sibling donor, we did a search and found a matched unrelated donor (MUD). He experienced graft rejection following HCT, a phenomenon previously associated with antitumor activity (33). In 2015, he was diagnosed with colorectal cancer and underwent radical resection. His postoperative course was complicated by recurrent episodes of septicemia, herpes zoster infection, skin cancer, anemia, osteoporosis, hyperferritinemia, and malabsorption. Despite these comorbidities, he remains alive and free of PDAC fifteen years after diagnosis.

## Discussion

The long-term survival observed in these three patients is remarkable, given the typically poor prognosis associated with PDAC. Pancreatic ductal adenocarcinoma remains one of the most lethal human malignancies and is currently the third leading cause of cancer-related death in the United States (34, 35). Extensive tumor heterogeneity and complex interactions between neoplastic epithelial cells and the surrounding tumor microenvironment are key features of PDAC biology, contributing to its dismal natural history (1). Although genetically diverse cancers may, in principle, respond to immunotherapy, all previous attempts in PDAC—including checkpoint inhibition and tumor-infiltrating lymphocyte (TIL) therapy—have failed to yield meaningful clinical results (2). Recent advances have identified actionable genetic alterations in a subset of PDAC patients; however, only a small fraction has benefited from molecularly targeted therapy (36).

Likewise, progress with various chemotherapy combinations has been minimal (10, 37). HCT exerts a well-documented antitumor effect, mediated by both acute and chronic GVHD (12, 13, 22). Beyond leukemia, this allogeneic anticancer effect has been demonstrated in several solid tumors, including renal cell carcinoma, colorectal carcinoma, and others (16, 17, 22, 24, 38). Similar allogeneic effects have been reported in PDAC, although none of those patients achieved long-term survival (19–22, 39).

In our study, all three patients underwent radical surgical resection, leaving minimal residual tumor burden and no distant metastasis—conditions that may favor an allogeneic anticancer response. Each patient experienced some degree of allogeneic effect following HCT. The first two developed both acute and chronic GVHD, which have been associated with antitumor activity (12, 13, 22). The third patient rejected the graft, and graft rejection itself has been linked to antitumor responses in both preclinical and clinical settings (33, 38). None of the three patients exhibited any signs of PDAC recurrence, as indicated by persistently low CA 19–9 levels and absence of detectable lesions on serial CT imaging.

Since all three patients had GVHD or rejection, which have anti-cancer effects (12, 13, 22, 23, 39), it may be important to induce anti-cancer effects following HCT in possible subsequent PDAC patients without GVHD. One such possibility is to give donor lymphocyte infusion, which gives an allogeneicanticancer effect, especially effective in HCT patients with chronic myeloid leukemia (40). Another possibility is to give low dose cyclosporine of short duration to try to induce mild acute GVHD and mild chronic GVHD. The best long-term survival was seen in leukemic patients who developed mild acute and chronic GVHD (41). We used this strategy in leukemic patients undergoing HCT and were able to induce mild acute and mild chronic GVHD and to improve survival (42, 43). This strategy may also enhance the antitumor effect in PDAC patients undergoing HCT as immunotherapy. We initially intended to expand the HCT trial to include additional patients with unrelated donors. However, the emergence of pharmaceutical company–driven chemotherapy trials redirected patient referrals away from HCT. With the advent of post-transplant cyclophosphamide–based immunosuppression and the broader use of HLA-haploidentical donors, it is now feasible to identify suitable donors for nearly all patients eligible for HCT (44, 45).

All three patients also developed secondary malignancies following HCT. The first patient developed oral cancer, which was successfully resected. The second, a heavy smoker and drinker, developed esophageal cancer that led to death nineteen years after PDAC diagnosis. The third patient developed both skin and colorectal cancers, which were also successfully treated. An increased risk of secondary malignancies has been well documented after transplantation, including HCT, compared with the general population (46, 47). Risk factors for secondary malignancies following HCT among others include immunosuppressive therapy and chronic GVHD (46, 47). However, patients receiving reduced-intensity conditioning (RIC) prior to HCT do not appear to have a higher risk of secondary malignancy than the general population, as shown in a multicenter study conducted by the Center for International Blood and Marrow Transplant Research (48). Despite receiving RIC, all three patients in the present study developed secondary cancers. Thus, we may speculate that these three individuals with PDAC may possess an intrinsic predisposition toward malignancy development.

Whipple’s surgery followed by HCT represents a highly demanding therapeutic course. Nevertheless, given the prolonged survival observed, the treatment appears justified, and the patients themselves expressed appreciation for the outcomes despite associated complications and side effects.

The key takeaway from these cases is that immunotherapy can be effective in PDAC when the tumor burden is minimal or undetectable. The graft-versus-leukemia (GVL) or more broadly GVT effect is comparable when using matched sibling donors (MSD), matched unrelated donors (MUD), or HLA-haploidentical donors (49, 50). Therefore, any suitably matched or partially matched donor may provide therapeutic benefit. The first two patients, who received grafts from MSD donors, developed both acute and chronic GVHD, suggesting that these immune effects may have eradicated residual PDAC cells (51). The third patient’s graft rejection likely also contributed to antitumor activity, as demonstrated in experimental models and supported by prolonged post-rejection survival in clinical HCT (33, 38).

All three patients had some sort of minimal residual disease, with no radiologic evidence of metastasis. In contrast, previous reports of HCT for PDAC have shown that all patients ultimately died from progressive disease despite evidence of GVT effects (19, 21, 22, 38). The recurrence in those cases likely reflected higher initial tumor burden compared to ours, emphasizing that immunotherapy is most effective in PDAC patients with very low or no measurable disease.

In conclusion, novel immunotherapeutic approaches—including checkpoint inhibitors, tyrosine kinase inhibitors, anti-matrix and anti-mitochondrial targeting agents, and other precision oncology strategies—may be considered for PDAC patients who have undergone radical surgery and exhibit low tumor burden (8, 10, 51–57). Such interventions may provide benefit when applied early, as in our three patients. Although all patients developed secondary malignancies (oral, esophageal, colorectal, and skin cancers)—a known risk following HCT (58). Their use of reduced-intensity conditioning likely mitigated the overall risk (48). The principal limitation of this study remains its small sample size. Nonetheless, we assume that these patients may have been cured of their PDAC. Of course, the risk of the procedure has to be taken into account, and it remains up to the patients to decide whether the side-effects weigh out the gain of lifetime. This gives hope to future immunotherapeutic approaches offering long-term tumor control in the adjuvant setting. However, its strength lies in the exceptionally long follow-up—from fifteen years to two decades—which suggests HCT or other types of immunotherapies as options for PDAC patients being radically operated. To our knowledge, such prolonged survival in PDAC has not been previously reported.

## Data Availability

The raw data supporting the conclusions of this article will be made available by the authors, without undue reservation.
